# Neurophobia among medical students and non-specialist doctors in Sri Lanka

**DOI:** 10.1186/1472-6920-13-164

**Published:** 2013-12-09

**Authors:** Anne Thushara Matthias, Poorna Nagasingha, Priyanga Ranasinghe, Saman B Gunatilake

**Affiliations:** 1 Department of Clinical Medicine, Faculty of Medical Sciences, University of Sri Jayewardenepura, Colombo, Sri Lanka; 2 Department of Pharmacology, Faculty of Medicine, University of Colombo, Colombo, Sri Lanka

**Keywords:** Neurophobia, Non-specialist doctors, Medical students, Sri Lanka

## Abstract

**Background:**

Neurophobia is the fear of neurosciences held by medical students and doctors. The present study aims to identify whether Neurology is considered a difficult subject by medical students and non-specialist doctors from Sri Lanka and evaluate reasons for such perceived difficulties.

**Methods:**

The study was conducted from May-June 2008. One hundred non-specialist doctors from the Colombo South Teaching Hospital and 150 medical students from the University of Sri Jayewardenepura were invited for the study. Data were collected by a pre-tested expert-validated self-administered questionnaire, designed to assess the degree of perceived difficulty, confidence, interest and knowledge of Neurology as compared to other subjects. It also evaluated reasons and probable strategies to overcome the perceived difficulties and/or lack of interests.

**Results:**

All non-specialist doctors and 148 medical students responded to the questionnaire (response rate–99.2%). The most favourite subject among medical students and non-specialist doctors were Cardiology and Endocrinology respectively, while Neurology was ranked third. In all participants the current level of interest was most for Cardiology (3.52±1.36), while Neurology was the least interesting specialty for majority of medical students (18.5%) and non-specialist doctors (25.0%). The current level of knowledge among medical students was most for Cardiology (3.12±0.86), while Neurology (2.53±0.96) was ranked fifth. The most difficult specialty for majority of medical students (50.0%) and non-specialist doctors (41.7%) was Neurology. All the participants were least confident when dealing with patients with headache (2.20±0.81), numbness of feet (2.07±0.79) and dizziness (2.07±0.78) when compared to dealing with other non-neurological complaints. The commonest reasons ‘why Neurology was felt to be a difficult subject’ were; the need to know basic neuro-anatomy and having a complex clinical examination. Participants’ felt that clinical/hospital based teaching (3.49±0.65), case discussions (3.45±0.68) and teaching aids (3.10±0.89) would be the most important teaching strategies to improve their competency in Neurology.

**Conclusion:**

Neurology is considered a difficult subject by undergraduates and non-specialist doctors of Sri Lanka. The main reason for the perceived difficulty was the lack of understanding of basic sciences and deficiencies in clinical teaching. This lack of confidence could have a significant impact on patient care.

## Background

The term ‘Neurophobia’ was originally coined by Jozefowicz to describe 'the fear of neural sciences and Neurology among medical students and non-specialist doctors’ [1]. In 2002, a study conducted in UK showed that Neurology was perceived by medical students’ as the most difficult of the sub-specialties [2]. Neurophobia is also very common among non-specialist doctors, especially amongst the General Practitioners (GPs) [2]. In addition, patients have also corroborated the view that non-specialist doctors' show lack of confidence and are unwilling to manage neurological diseases [3]. Hence, it is evident that neurophobia is a global issue, with implications on patient care and satisfaction [4]. The exact reasons for neurophobia are not well known and it likely stems as a result of multiple contributory factors. The manner in which neuroscience and Neurology are taught, its’ complex and sometimes abstract subject matter, and the length of time that must be devoted in order to elicit clinical signs are likely factors [5]. The fact that neurologists themselves enjoy the perceived notion that theirs is a difficult subject only suited for the most brilliant is further confounding matters [2]. According to World Health Organization (WHO) estimates, neurological conditions contribute approximately 6.3% to the global health burden [6]. In addition, the prevalence and public health impact of neurological diseases is rising globally due to the ageing of the worlds’ population [7].

Sri Lanka is a middle income South Asian country with a population of nearly 21 million. Cerebro-vascular disease is a leading cause of adult disability in Sri Lanka and it is the fifth leading cause of hospital death [8]. Hospital admissions due to Cerebrovascular diseases have increased by about 20% from 170,000 in 1999 to 210,000 in 2005 [8]. In addition other neurological disorders such as epilepsy, Parkinson’s disease and migraine are common in Sri Lanka and their prevalence is not different from other countries. Neurology is increasingly becoming an outpatient-based service in Sri Lanka; people with chronic neurological disorders such as strokes are now often being managed in the community. Hence general practitioners need to be comfortable in dealing with neurological disorders. The increasingly multidisciplinary nature of patient care means that hospital-based doctors from other specialties should also be comfortable in dealing with neurological problems. There are only about 30 certified neurologists serving the country at present, with the demand far exceeding the requirement. Hence, more patients will have to rely on general practitioners and non-specialist doctors in hospitals for their neurological care. Therefore, it is imperative that Sri Lankan non-specialist doctors and medical students who will become future doctors are self-assured and proficient in the diagnosis and management of neurological disease.

Current status of knowledge and clinical competency in Neurology of final year students and junior doctors are critical information for those involved in development of medical curricula. Studies are required to identify the extent of the problem. At present no data exists on the perceived competence and knowledge on Neurology amongst medical students and non-specialist doctors from Sri Lanka. As neurophobia appears to be a problem seen world over, we were interested in assessing the situation in our medical school and hospital. The present study aims to evaluate the attitudes of medical students and non-specialist doctors toward Neurology and neurological education with respect to perceived interest, difficulty, knowledge and confidence compared to other medical specialties. We also aim to identify the reasons and sources for the perceived difficulty and probable strategies to overcome them, while providing data that could help design curricula for a more effective education in Neurology.

## Methods

The study was conducted from May to June 2008 at the Faculty of Medical Sciences, University of Sri Jayewardenepura (USJP), Sri Lanka and the Colombo South Teaching Hospital (CSTH), Colombo, Sri Lanka. The CSTH is the second largest government hospital in the Colombo district, with a bed strength of nearly 1,100. Annually, it provides treatment to about 150,000 inward patients and 750,000 out patients [9]. The Faculty of Medical Science, USJP admits around 150 students each year to follow the five year MBBS degree programme [10]. All non-specialist doctors (intern house officers and senior house officers) working at CSTH during the study period were invited for the study. The non-specialist doctors were from the following specialties; Anesthesia, Cardiology, Dermatology, Endocrinology, ENT surgery, Eye surgery, Gastroenterology, Genitor urinary surgery, Gynecology and Obstetrics, Medicine, Orthopedics, Pediatrics, Psychiatry, Radiology, Rheumatology and Surgery. One hundred and fifty final year medical students from a single batch who were doing clinical appointments in the final year at the CSTH were also invited. They were contacted by visiting the respective wards during the study period. Ethical and institutional approval for the study was obtained from the Ethics Review Committee at CSTH.

Data were collected by a pre-tested expert-validated self-administered questionnaire (Additional file 1). The questionnaire was designed based upon questionnaires used successfully in other similar studies [2,11]. A panel of experts consisting of neurologists and medical educators evaluated the contents of the questionnaire. The questionnaire was pre-tested among 10 doctors and 10 medical students who were not taking part in the study. The suggested changes during expert-validation and pre-testing was carried our prior to the commencement of the study. The questionnaire was designed to assess the degree of perceived difficulty, confidence, interest and knowledge of Neurology as compared to other subjects. In the questionnaire the following seven specialties were considered; Cardiology, Dermatology, Endocrinology, Gastroenterology, Neurology, Respiratory Medicine and Rheumatology. The participants were asked to; a) rank each of the above subjects on a likert scale of ‘0’ – least favourite to ‘5’ – most favourite, b) rate their current level of interest in each of the above subjects on a likert scale of ‘0’ – not interested to ‘5’ – very interested and c) organize the above subjects in a hierarchy from least interesting to most interesting. Thereafter the participants were asked to rate their current level of knowledge on each of the above subjects on a likert scale of ‘1’ – very poor to ‘5’ – very good. This was followed by a likert scale question evaluating perceived difficulty of each subject (‘1’ – very easy to ‘5’ – very difficult). The respondents were also asked to rank the above subjects from least to most difficult. The seventh question was a likert scale question inquiring about the level of confidence (‘0’ – no confidence to ‘4’ – highly confident) in managing the following common patient complaints related to each of the above systems; abdominal pain, chest pain, cough, dizziness, fever, headache, heartburn, numbness of feet and shortness of breath.

The remaining sections of the questionnaire focused on reasons and probable strategies to overcome the perceived difficulties and/or lack of interest. They were asked to rank on a likert scale (‘0’ – Strongly disagree to ‘4’ – Strongly agree) ‘why Neurology was felt to be a difficult subject’. The following options were provided 1) the need to know basic neuro-anatomy, 2) having a complex clinical examination, 3) having a large number of complex and rare diagnosis, 4) having a reputation of being difficult, 5) being poorly taught, 6) not having enough teaching time, 7) not having a definitive curative treatment in most instances and 8) being a complex subject. In the last question the participants were asked to rate the following teaching strategies that would help to improve Neurology competency on a likert scale (‘0’ – Strongly disagree to ‘4’ – Strongly agree); a) clinical/hospital based teaching, b) Neurology lectures, c) neuro-anatomy lectures, d) case discussions and e) teaching aids.

The data were analyzed using SPSS v14 for windows (SPSS Inc., Chicago, IL, USA) statistical software package. The answers of the Likert score were tabulated in to mean scores. The comparison and significance was done using one way ANOVA and a p < 0.05 was considered to be statistically significant.

## Results

One hundred non-specialist doctors and 148 medical students responded to the questionnaire (response rate – 99.2%). Among medical students the most favourite subject was Cardiology with a mean ± SD of 4.71 ± 2.32, followed by Endocrinology (4.10 ± 1.81), Respiratory Medicine (4.03 ± 1.68) and Neurology (3.93 ± 1.99). The mean for Neurology among medical students were significantly different from the means for Respiratory Medicine, Rheumatology and Gastroenterology (p < 0.01). Among doctors Endocrinology (mean ± SD) was the most favourite (4.33 ± 1.73), followed by Cardiology (4.18 ± 2.17), Neurology (4.14 ± 2.17) and Dermatology (4.04 ± 2.30). The mean for Neurology among the doctors were significantly higher than the mean for Dermatology and Gastroenterology (p < 0.05). In all participants the level of interest was most for Cardiology with a mean ± SD of 3.52 ± 1.36 and Endocrinology was second with a mean ± SD of 2.86 ± 1.32, followed by Neurology with a mean ± SD of 2.83 ± 1.39. There was no difference in this order between medical students and non-specialist doctors. The mean level of interest for Neurology was significantly different from the means of all other specialties (p < 0.01).

**Figure 1:**
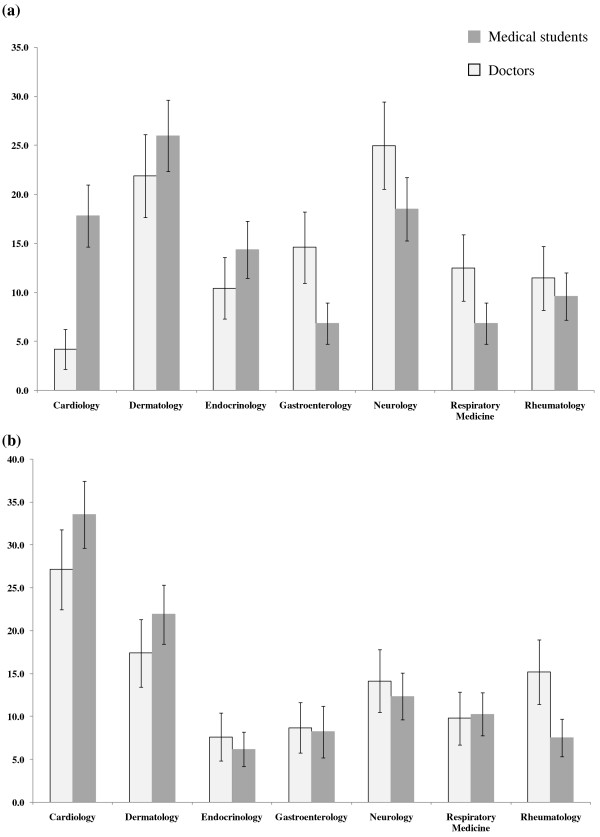
Preference for the different medical specialties among medical students and doctors a) least interested specialty and b) most interested specialty.

When respondents were asked to rank the seven medical specialties according to the level of interest from least interested to most interested, Neurology was the least interesting specialty for 18.5% of medical students and 25.0% of non-specialist doctors (Figure 1a). The most interesting specialty for majority of the medical students (33.6%) and non-specialist doctors (25.0%) was Cardiology (Figure 1b). The current level of knowledge among medical students on the above specialties was highest for Cardiology with a mean ± SD of 3.12 ± 0.86, followed by Respiratory Medicine (3.00 ± 0.90), while Neurology (2.53 ± 0.96) was ranked fifth. A similar pattern was also observed amongst the non-specialist doctors (Table 1). The perceived level of difficulty on a likert scale from 1 to 5 was significantly higher for Neurology (mean ± SD) than other specialties among both medical students (3.46 ± 0.99) and non-specialist doctors (3.33 ± 1.11) (Table 1). The most difficult specialty for majority of medical students (50.0%) and non-specialist doctors (41.7%) was Neurology.

**Table 1:** Current level of knowledge, difficulty and confidence in the different medical specialties among medical students and non-specialist doctors

	**Mean score (±SD)**	
	**Medical Students (n = 148)**	**Doctors (n = 100)**
Current level of knowledge (range 0–5)		
Cardiology	3.12 (±0.86)^*^	2.96 (±1.03)^*^
Respiratory Medicine	3.00 (±0.90)^*^	2.86 (±1.00)^*^
Gastroenterology	2.84 (±0.87)^*^	2.75 (±0.90)^*^
Endocrinology	2.54 (±0.99)	2.59 (±0.98)
Neurology	2.53 (±0.96)^*^	2.59 (±1.08)^*^
Rheumatology	2.42 (±0.94)	2.57 (±1.06)
Dermatology	2.16 (±1.00)^*^	2.49 (±1.10)
Level of difficulty (range 0–5)		
Neurology	3.46 (±0.99)^*^	3.33 (±1.11)^*^
Endocrinology	3.07 (±0.96)^*^	3.03 (±0.90)^*^
Cardiology	3.06 (±0.84)^*^	3.03 (±0.98)^*^
Gastroenterology	2.84 (±0.78)^*^	2.55 (±0.87)^*^
Rheumatology	2.82 (±0.88)^*^	2.64 (±1.08)^*^
Dermatology	2.80 (±1.11)^*^	2.76 (±0.90)^*^
Respiratory Medicine	2.73 (±0.82)^*^	2.69 (±0.93)^*^
Level of confidence (range 0–4)		
Shortness of breath	2.72 (±0.66)^a,b,c^	2.58 (±0.80)^a,b,c^
Chest pain	2.70 (±0.75)^a,b,c^	2.66 (±0.85)^a,b,c^
Fever	2.68 (±0.73)^a,b,c^	2.80 (±0.79)^a,b,c^
Heartburn	2.65 (±0.72)^a,b,c^	2.91 (±0.83)^a,b,c^
Cough	2.62 (±0.65)^a,b,c^	2.94 (±0.72)^a,b,c^
Abdominal pain	2.35 (±0.69)^a,b,c^	2.45 (±0.79)^a,b,c^
Headache	2.13 (±0.78)^a^	2.30 (±0.86)^a^
Numbness of feet	2.03 (±0.64)^b^	2.13 (±0.78)^b^
Dizziness	1.91 (±0.73)^c^	2.31 (±0.79)^c^

^*^Mean for Neurology significantly different from means for these specialties (p < 0.01); ^a,b,c^Neurological complaints had a significantly lower mean compared with other complaints (p < 0.05)

Regarding the level of confidence in managing common patient complaints, all the participants were least confident when dealing with headache with a mean ± SD of 2.20 ± 0.81, numbness of feet (2.07 ± 0.79) and dizziness (2.07 ± 0.78) when compared to all other non neurological complaints (p < 0.001). A similar pattern was observed amongst the medical students and non-specialist doctors when considered separately (Table 1). In all participants the commonest reasons ‘why neurology was felt to be a difficult subject’ were; a) the need to know basic neuro-anatomy (mean ± SD – 3.09 ± 0.98), b) complex clinical examination (mean ± SD – 2.80 ± 0.96), c) being a complex subject (mean ± SD – 2.74 ± 1.01) and d) having large number of complex and rare diagnosis (mean ± SD – 2.59 ± 1.02). The common reasons were similar in both medical students and non-specialist doctors (Table 2). Both the students and the non-specialist doctors felt that clinical/hospital based teaching (mean ± SD – 3.49 ± 0.65), case discussions (mean ± SD – 3.45 ± 0.68) and teaching aids (mean ± SD – 3.10 ± 0.89) were the most important teaching strategies to improve competency in Neurology (Table 3).

**Table 2:** Reasons for Neurology being perceived as a difficult subject

	**Mean Score (±SD)**	
	**Medical Students**	**Non-specialist Doctors**
The need to know basic neuro-anatomy	3.05 (±1.04)	3.15 (±0.91)
Being a complex subject	2.79 (±1.02)	2.67 (±1.00)
Having a complex clinical examination	2.69 (±1.04)	2.98 (±0.78)
Having large number of complex and rare diagnosis	2.60 (±1.09)	2.57 (±0.92)
Not having enough teaching time	2.57 (±1.12)	2.23 (±1.19)
Being poorly taught	2.20 (±1.19)	2.13 (±1.22)
Having a reputation of being difficult	2.14 (±1.16)	1.71 (±0.99)
Not having definitive curative treatment in most instances	1.93 (±1.14)	1.77 (±1.12)

**Table 3:** Teaching strategies to improve competency in Neurology

	**Mean score (±SD)**	
	**Medical Student**	**Non-specialist Doctors**
Clinical/hospital based teaching	3.46 (±0.71)	3.53 (±0.55)
Case discussions	3.39 (±0.72)	3.53 (±0.62)
Teaching aids	3.17 (±0.86)	3.08 (±0.94)
Neurology lectures	3.13 (±0.84)	3.04 (±0.90)
Neuro-anatomy lectures	2.90 (±0.99)	2.92 (±0.82)

## Discussion

Around 10-20% of all acute hospital admissions are neurological illnesses or related to neurology [12]. Over the last few decades the number of neurology related illnesses presenting to hospitals has doubled from 134.1 per 100,000 population in 1985 to 274.7 per 100,000 population in 2006 [13]. Most neurological illnesses and complaints are not managed by specialist Neurologist but rather by GPs and physicians [14]. Therefore it is important that medical students who will be future doctors have a sound knowledge of Neurology and are not anxious when dealing with neurological problems. Several studies from UK and Ireland have reported that students and junior doctors find Neurology to be the most difficult subject amongst the different medical specialties [2]. At present there are no data on the presence and extent of Neurophobia among students and doctors from developing South Asian countries. Our results show that Sri Lankan medical students and non-specialist doctors consider neurology to be a difficult subject and have the least interest, knowledge and confidence in managing neurological complaints.

One of the main reasons why Neurology was considered difficult was the lack of knowledge in basic neurosciences and neuro-anatomy. This is a problem that could be remedied by vertical integration of basic neurosciences and clinical training. It was evident from our results that subjects were least confident when dealing with neurologically related complaints. This could be the result of having low level of knowledge, lack of confidence in nervous system examination and perceiving Neurology to be difficult. When participants were asked why Neurology was considered to be difficult two factors stood out: the need to know basic neurosciences and complexity of the clinical examination. One way of overcoming this would be to reinforce the knowledge of basic neuro-anatomy and physiology during the clinical teaching sessions, where students would be in a better, mature position to apply their basic science knowledge to clinical situations.

In a study on the effects of neurophobia on clinical practice in the United Kingdom [15], the two main findings were that neurological diseases are increasing in number in the UK and general practitioners lack confidence in dealing with them, resulting in an increased number of referrals to specialists. This is probably true if tested in Sri Lanka, as the students and doctors were least confident when dealing with symptoms associated with Neurology. The solution to this phobia is to improve the undergraduate and postgraduate medical curricula in Neurology to make it more learner friendly. Case-based teaching in Australia and teaching videos in Singapore have met with some success [16,17]. According to our study Neurology was a subject in which the participants were interested, as it ranked third amongst the interest shown in subjects, a finding that has also been seen in several previous studies [2,5].

Sri Lanka is served by about 30 neurologists (one neurologist per 600,000 people) [8]. As a result Neurology as a specialty is available only in a limited number of hospitals and most junior doctors would not be exposed to seeing and managing Neurology patients under proper guidance. The present curriculum at Faculty of Medical Sciences, University of Sri Jayewardenepura is an integrated system based curriculum spanning over three phases. Phase I (first 2 years) consists of five terms which includes ten modules, of which the “Neurosciences” module (4 weeks) deals with the basic knowledge on development, structure and function of the nervous system. Phase II (3^rd^ and 4^th^ years) consists of sixteen modules, of which the “Neurology & Musculoskeletal Module” (4 weeks) focuses on the different neurological disorders. In the fourth year students attend a Neurology clinical attachment for two weeks and also see a variety of Neurology patients in the medical wards. Hence, the reason for neurophobia in Sri Lanka appears to be not the lack of interest and opportunity, but deficiencies in current teaching and learning methods.

Future studies should aim to identify strategies to decrease neurophobia in medical students and doctors. A recent systematic review by McColgan, et al on educational interventions in Neurology, concluded that there is very little high quality evidence for demonstrably effective Neurology education interventions [18]. Recent evidence suggests that, at least at medical school, staff development linked to student assessment of teaching can reduce neurophobia [15]. There is also evidence that educational strategies such as supported participation and video-recordings can also improve Neurology education and reduce neurophobia [19,20]. Hence there is also an urgent need to improve the evidence base for quality Neurology education interventions that will ultimately help to reduce neurophobia.

We would like to highlight several limitations in the present study. The study was performed at a single medical faculty and teaching hospital in Sri Lanka. Currently there are 8 medical faculties and over 20 teaching hospitals in Sri Lanka. Hence, our findings may be difficult to be generalized to other medical schools in the Sri Lanka, where variability in curricula and teaching methods may lead to different observations. However, similar trends have been observed in many other studies in Europe and US, suggesting that the issues identified may be common amongst educational establishments even with variations in curricula [2,11,16]. In addition we only evaluated the perception, knowledge, interest and confidence of medical students and non-specialist doctors’ with regards to Neurology. Further studies are needed to determine if the perceived difficulties correlate with poor performance at examinations and in compromised patient care.

## Conclusion

Neurology is considered difficult by the undergraduates and the non-specialist doctors in Sri Lanka. The main reason for the perceived difficulty was the lack of understanding of basic sciences. This lack of confidence could hinder participants selecting Neurology as a specialty in the future. Even in the event of becoming a general practitioner, the lack of confidence could have a significant impact on patient care. Current undergraduate and postgraduate teaching programmes should take these observations into account to amend their curricula to correct these deficiencies.

## Competing interests

The author(s) declare that they have no competing interests.

## Authors’ contributions

ATM, PN, and SBG made substantial contribution to conception and study design. ATM and PN were involved in data collection. ATM, PN and PR were involved in refining the study design, statistical analysis and drafting the manuscript. PR and SBG critically revised the manuscript. All authors read and approved the final manuscript.

## Pre-publication history

The pre-publication history for this paper can be accessed here:

http://www.biomedcentral.com/1472-6920/13/164/prepub

## Supplementary Material

Additional file 1 Data collection questionnaire. Click here for file
