# Supplementary material for: Neurophobia among medical students and non-specialist doctors in Sri Lanka
Source: BMC Med Educ. 2013 Dec 9;13:164. doi: 10.1186/1472-6920-13-164 (PMC3909313; doi:10.1186/1472-6920-13-164)
Supplement: Additional file 1 — Data collection questionnaire. [file 1472-6920-13-164-S1.pdf]

Study ID: ☐☐☐☐☐☐☐

Questionnaire

Date of Birth : ☐☐☐☐☐☐☐☐ (1)
d d m m y y y y

Gender : Male [1] Female [2]

Are you a, Doctor ☐ Medical Student ☐

Q1 What is your level of preference for each of the following specialties?
(Please circle the appropriate 1-5)

	Least favourite				Most favourite
Cardiology	1	2	3	4	5
Dermatology	1	2	3	4	5
Endocrinology	1	2	3	4	5
Gastroenterology	1	2	3	4	5
Nephrology	1	2	3	4	5
Neurology	1	2	3	4	5
Respiratory medicine	1	2	3	4	5
Rheumatology	1	2	3	4	5

Q2 What is your current level of interest in each of the following specialties?
(Please circle the appropriate level of interest 1-5)

	Not interested				Very interested
Cardiology	1	2	3	4	5
Dermatology	1	2	3	4	5
Endocrinology	1	2	3	4	5
Gastroenterology	1	2	3	4	5
Nephrology	1	2	3	4	5
Neurology	1	2	3	4	5
Respiratory medicine	1	2	3	4	5
Rheumatology	1	2	3	4	5

Q3 List below mentioned subjects from least interesting (1) to most interesting (7)
(Cardiology, Dermatology, Endocrinology, Gastroenterology, Neurology, Respiratory medicine, Rheumatology)

1 ______________________

2 ______________________

3 ______________________

4 ______________________

5 ______________________

6 ______________________

7 ______________________

Study ID:

Q4 What is your current level of knowledge in each of the following specialties?
(Please circle the appropriate level of knowledge 1-5)

	Very low				Very high
Cardiology	1	2	3	4	5
Dermatology	1	2	3	4	5
Endocrinology	1	2	3	4	5
Gastroenterology	1	2	3	4	5
Nephrology	1	2	3	4	5
Neurology	1	2	3	4	5
Respiratory medicine	1	2	3	4	5
Rheumatology	1	2	3	4	5

Q5 What is your perceived level of difficulty in each of the following specialties?
(Please circle the appropriate level of difficulty 1-5)

	Very easy				Very difficult
Cardiology	1	2	3	4	5
Dermatology	1	2	3	4	5
Endocrinology	1	2	3	4	5
Gastroenterology	1	2	3	4	5
Nephrology	1	2	3	4	5
Neurology	1	2	3	4	5
Respiratory medicine	1	2	3	4	5
Rheumatology	1	2	3	4	5

Q6 List below mentioned subjects from least difficult (1) to most difficult (7)
(Cardiology, Dermatology, Endocrinology, Gastroenterology, Neurology, Respiratory medicine, Rheumatology)

1 ______________________

2 ______________________

3 ______________________

4 ______________________

5 ______________________

6 ______________________

7 ______________________

Study ID:

Q7 What is your current level of confidence in managing each of the following?
(Please circle the appropriate level of confidence 1-5)

	No confidence				Highly confident
Abdominal Pain	1	2	3	4	5
Chest Pain	1	2	3	4	5
Cough	1	2	3	4	5
Dizziness	1	2	3	4	5
Fever	1	2	3	4	5
Headache	1	2	3	4	5
Heartburn	1	2	3	4	5
Numbness of feet	1	2	3	4	5
Shortness of breath	1	2	3	4	5

Q8 If your most difficult subject is neurology,
Why neurology was felt to be a difficult subject?
(Please circle the appropriate from 1-4)

	Strongly disagree			Strongly agree
1 The need to know basic neuro-anatomy	1	2	3	4
2 Having a complex clinical examination	1	2	3	4
3 Having large number of complex and rare diagnosis	1	2	3	4
4 Having a reputation of being difficult	1	2	3	4
5 Being poorly taught	1	2	3	4
6 Not having enough teaching time	1	2	3	4
7 Not having definitive curative treatment in most instances	1	2	3	4
8 Neurology being a complex subject	1	2	3	4

Q9 Do you feel that the following teaching strategies
will help to improve your competency in neurology?
(Please circle the appropriate from 1-4)

	Strongly disagree			Strongly agree
1 Clinical/hospital based teaching	1	2	3	4
2 Neurology lectures	1	2	3	4
3 Neuro-anatomy lectures	1	2	3	4
4 Case discussions	1	2	3	4
5 Teaching aids	1	2	3	4
